# ALPAR: automated learning pipeline for antimicrobial resistance

**DOI:** 10.1093/bioinformatics/btag545

**Published:** 2026-07-22

**Authors:** Alper Yurtseven, Roman Joeres, Olga V Kalinina

**Affiliations:** Department of Drug Bioinformatics, Helmholtz Institute for Pharmaceutical Research Saarland (HIPS), Helmholtz Centre for Infection Research (HZI), Saarbrücken, Saarland 66123, Germany; Center for Bioinformatics, Saarland University, Saarbrücken, Saarland 66123, Germany; Department of Drug Bioinformatics, Helmholtz Institute for Pharmaceutical Research Saarland (HIPS), Helmholtz Centre for Infection Research (HZI), Saarbrücken, Saarland 66123, Germany; Center for Bioinformatics, Saarland University, Saarbrücken, Saarland 66123, Germany; Department of Drug Bioinformatics, Helmholtz Institute for Pharmaceutical Research Saarland (HIPS), Helmholtz Centre for Infection Research (HZI), Saarbrücken, Saarland 66123, Germany; Center for Bioinformatics, Saarland University, Saarbrücken, Saarland 66123, Germany; Faculty of Medicine, Saarland University, Saarland 66421, Germany

## Abstract

**Summary:**

The field of machine learning in antimicrobial resistance (AMR) research has experienced rapid growth, fueled by advancements in high-throughput genome sequencing and the growing capacity of computational resources. However, the complexity and lack of standardized data preparation and bioinformatic analyses present significant challenges, especially for newcomers to the domain. In response to these challenges, we introduce ALPAR (Automated Learning Pipeline for Antimicrobial Resistance), a comprehensive AMR data analysis tool covering the entire process from processing of raw genomic data to training machine learning models to interpretation of results. Our method relies on a reproducible pipeline that integrates widely used bioinformatics tools, presenting a simplified, automatic workflow specifically tailored for single-reference AMR analysis. Accepting genomic data in the form of FASTA files as input, ALPAR facilitates the generation of machine learning-ready data tables and both the training of machine learning and the execution of genome-wide association studies (GWAS) experiments. Additionally, our tool offers supplementary functionalities such as phylogeny-based analysis of the distribution of mutations, enhancing its utility for researchers. The tool has also proven its performance in competitive benchmarks, winning the 2024 CAMDA Anti-Microbial Resistance Prediction Challenge and placing third in the 2025 edition.

**Availability and implementation:**

ALPAR is open-source and freely accessible via GitHub (https://github.com/kalininalab/ALPAR). The pipeline is fully reproducible and can be easily installed as a Conda package (https://anaconda.org/kalininalab/ALPAR)

## 1 Introduction

Antimicrobial resistance (AMR) is a pressing global health concern, posing significant challenges to the effective treatment of bacterial infections and jeopardizing decades of progress in modern medicine (World Health Organization 2014, O’Neill 2016, CDC 2019). The rapid evolution and spreading of antimicrobial-resistant pathogens have necessitated innovative approaches to understand, monitor, and combat this growing threat (Ventola 2015, Holmes *et al.* 2016).

Recently, machine learning-based methodologies have emerged as powerful tools in the study of AMR, capitalizing on advancements in high-throughput DNA sequencing technologies to elucidate complex relationships between genomic mutations and AMR; a well-known example of such a relationship is the single-nucleotide polymorphisms (SNPs) in the *gyrA* gene, which cause resistance towards ciprofloxacin (Khaledi *et al.* 2020, Ren *et al.* 2022, Tang *et al.* 2022). The application of machine learning algorithms in AMR research holds promise for revolutionizing our understanding of bacterial resistance mechanisms and informing more targeted therapeutic interventions. By systematically analyzing vast datasets comprising genomic sequences and AMR phenotypic profiles, these algorithms can uncover subtle patterns and associations that may escape traditional analytical methods (Paredes-Gutierrez *et al.* 2025).

Traditionally, AMR prediction from genomic data relies on rule-based approaches and genome-wide association studies (GWAS). Rule-based approaches involve applying predefined sets of rules derived from clinical guidelines (Zankari *et al.* 2012, McArthur *et al.* 2013). These approaches rely on predefined criteria to identify patterns in genomic sequences or clinical data linked to resistance to specific antimicrobial agents. For instance, certain genetic mutations or the presence of resistance genes (e.g. *bla* genes for beta-lactam resistance (Ranjbar and Sami 2017)) can be flagged as indicators of resistance. However, this method requires extensive and detailed knowledge of specific resistance mechanisms and frequent manual updates, making it challenging to apply across the diverse organisms encountered in clinical practice (Pesesky *et al.* 2016, Anahtar *et al.* 2021). They also often struggle to predict complex resistance patterns with multiple mechanisms involved, such as carbapenem resistance (Pesesky *et al.* 2016, Logan and Weinstein 2017, Sakagianni *et al.* 2024).

GWAS enhances this analysis by scanning large collections of bacterial genomes to identify genetic variants associated with resistance to antimicrobial agents (Mosquera-Rendon *et al.* 2023). GWAS compares the genomes of resistant and susceptible strains to pinpoint SNPs or other genetic markers that correlate with resistance (Weber *et al.* 2021). This approach enables the discovery of novel resistance mechanisms and genes, providing insights into the genetic basis of AMR (Chewapreecha *et al.* 2014, Farhat *et al.* 2016). GWAS is a powerful tool for understanding how resistance evolves and spreads, aiding in the development of new diagnostic tools and therapeutic strategies to combat resistant infections (Mosquera-Rendon *et al.* 2023). Therefore, combining rule-based methods and GWAS can offer a comprehensive approach to tackling antimicrobial resistance (Read and Massey 2014, Chen and Shapiro 2015, Power *et al.* 2017).

Machine learning (ML) approaches, on the other hand, offer the potential to accelerate the discovery of novel resistance markers, optimize treatment regimens, and mitigate the spread of resistant pathogens (Kim *et al.* 2022). Recent comprehensive reviews highlight this pattern shift, emphasizing how supervised learning is increasingly relied upon to translate complex genomic features into clinically actionable susceptibility profiles (Kim *et al.* 2022). Unlike traditional rule-based systems, which depend on predefined rules and can be limited by their static nature, ML algorithms can dynamically adapt to new data and uncover complex patterns that are not immediately apparent (Sakagianni *et al.* 2023).

In contrast to GWAS, ML methods are capable of identifying non-linear effects and interactions between multiple genetic markers (Elgart *et al.* 2022). Additionally, ML can integrate and analyze large-scale, multidimensional data from diverse sources, providing a more nuanced understanding of resistance mechanisms (Ardila *et al.* 2025).

This advanced analytical power enables the development of more accurate and personalized diagnostic tools and therapeutic strategies, significantly enhancing our ability to combat resistant infections and address the evolving challenges of antimicrobial resistance (Zou *et al.* 2025). ML has previously been applied in the AMR research. Traditional ML algorithms, such as support vector machines (SVMs) (Cortes and Vapnik 1995), have demonstrated strong predictive performance when applied to features such as single SNPs, gene expression profiles, and gene presence–absence (GPA) patterns in *Pseudomonas aeruginosa* (Khaledi *et al.* 2020). This foundational work by Khaledi *et al.* established the high predictive value of combining diverse genomic and transcriptomic signatures to accurately predict resistance phenotypes, enlightening the way for more advanced modeling in the field.

In addition, ensemble-based methods, particularly random forests (Breiman 2001), have also yielded promising results in AMR prediction tasks across various bacterial species (Ren *et al.* 2022).

Furthermore, gradient-boosted decision trees (GBDTs) have emerged as highly effective models in this domain, successfully predicting antibiotic resistance profiles from large-scale pan-genome data with high accuracy (Moradigaravand *et al.* 2018). Specifically, implementations like XGBoost have been extensively leveraged to predict categorical resistance and quantitative minimum inhibitory concentrations (MICs) utilizing both pan-genome subsets and reference-free *k*-mer profiles, often without relying on knowledge of resistance mechanisms (Nguyen *et al.* 2019, Wu *et al.* 2022). Beyond tree-based ensembles, the field is also rapidly adopting deep learning architectures such as convolutional neural networks (CNNs) and deep autoencoders to extract complex, non-linear sequence motifs directly from high-dimensional genomic data, enabling highly sensitive multidrug-resistance classification (Yang *et al.* 2019, Kim *et al.* 2022, Kulkarni *et al.* 2026).

However, standard ML approaches face a significant challenge when applied to bacterial genomics: they generally assume that training samples are independent and identically distributed (IID). Because bacterial populations are highly structured and sampling is often biased, naive ML models are prone to conflating phylogenetic lineage markers with genuine indicators of AMR. In this specific regard, ML methods have traditionally been inferior to GWAS approaches, which can successfully mitigate phylogenetic effects by explicitly correcting for population structure using mixed-effect models (Yu *et al.* 2025). Overcoming this lineage association bias remains a major challenge for ensuring the generalizability of ML-based AMR predictions. To address this limitation, we integrate a Phylogeny-Related Parallelism Score (PRPS) (Yurtseven *et al.* 2023) to penalize lineage-specific passenger mutations and leverage similarity-aware data splitting via DataSAIL (Joeres *et al.* 2025), ensuring that predictive performance is evaluated across phylogenetically distinct clades.

Beyond these algorithmic limitations, several practical challenges also obstruct the widespread adoption of ML in AMR research. A notable barrier is the extent of data preparation and bioinformatic analyses required for meaningful interpretation of genomic data in the context of AMR (Chindelevitch *et al.* 2022). Researchers, particularly those new to the field, often encounter difficulties navigating the numerous tools, formats, and protocols necessary for conducting robust AMR studies.

To address these limitations, we present ALPAR (Automated Learning Pipeline for Antimicrobial Resistance). The novelty of ALPAR lies in its architecture as a streamlined, end-to-end platform that transforms a traditionally fragmented and highly complex bioinformatic workflow into a unified, single-command executable. Rather than requiring researchers to manually bridge disparate software, ALPAR automatically integrates raw variant calling (SNPs and indels) with gene presence–absence (GPA) features to capture both high-resolution genomic variation and broader functional differences (James *et al.* 2025). By providing a standardized, ML-ready framework natively equipped with advanced dataset-splitting and feature-scoring capabilities, ALPAR significantly lowers the barrier to entry while elevating the precision of computational AMR studies.

## 2 Results

### 2.1 Design and implementation

ALPAR (Automated Learning Pipeline for Antimicrobial Resistance) is a software package implemented in Python for easy readability and extendability. It integrates several open-source bioinformatics tools into a unified single-reference AMR workflow.

The tool is built using a command-line interface (CLI) submodule architecture managed through argparse subparsers. This layout separates the computational workflow into self-contained modules, including variant calling and gene presence/absence submodules (create_binary_tables), a binary table thresholding module, a phylogenetic tree module (PanACoTA or MashTree), GWAS analysis, a Phylogeny Related Parallelism Score (PRPS) calculation module, and machine learning classification. Each submodule handles isolated steps of the pipeline independently, allowing users to either run individual stages or execute the end-to-end workflow seamlessly using the main automatix call.

The pipeline accepts genomic FASTA files and a reference genome as input. To generate binary mutation tables (for examples see Supplementary File 2, available as supplementary data at *Bioinformatics* online), ALPAR employs the following pipeline: Snippy (Seemann 2015) is used to call variants for each strain against a reference genome, Prokka (Seemann 2014) annotates each genome using a user-defined protein database, or alternatively Bakta (Schwengers *et al.* 2021) can be selected by the user for the annotation step, CD-HIT clusters annotated genomes to determine GPA information with minimal resource requirements, and Panaroo (Tonkin-Hill *et al.* 2020) provides more detailed GPA information. For phylogenetic analysis, ALPAR offers both alignment-free (using MashTree) and alignment-based (using PanACoTA) options (Katz *et al.* 2019, Perrin and Rocha 2021). To enhance model robustness, ALPAR incorporates phylogeny-related parallelism scores (PRPS) to prioritize functionally relevant genetic markers (Yurtseven *et al.* 2023) and employs DataSAIL to mitigate information leakage via similarity-aware data splitting (Joeres *et al.* 2025). Finally, the pipeline trains several ML models (RF, SVM, gradient boosting and fully regularized logistic regression with L1/L2 penalties) depending on the user’s configuration with a grid search method for easy parameter optimization.

For comprehensive model evaluation, ALPAR’s machine learning submodule automatically calculates and outputs performance metrics for every trained model, including ROC-AUC, precision, recall, accuracy, F1-score, and detailed confusion matrices, alongside the Matthews correlation coefficient (MCC) (Supplementary File 5, Figs. 5–6, available as supplementary data at *Bioinformatics* online).

To ensure broad applicability, ALPAR is designed with minimal operational dependencies. The pipeline’s primary dependency lies in the quality of the input genomic FASTA files and the chosen reference genome. Crucially, its architecture is independent of curated, species-specific resistance databases; unlike traditional rule-based approaches, which allow ALPAR to be deployed out-of-the-box for any bacterial species. To guarantee robustness against poor-quality input, ALPAR features an automated quality control (QC) module. The QC step checks the input genome’s quality by comparing its size with the given reference genome. Thresholds are mathematically defined and fully adjustable via the command line: contig count (qc_max_contigs) excludes genomes exceeding the maximum limit (default: 500), and length variance (qc_length_threshold) excludes genomes if their length deviates beyond a fraction (default: 0.1, or ±10%) of the reference length. The overall robustness of this integrated architecture has been validated across 16 unique bacterial species sourced from five distinct public repositories and one internal dataset, consistently executing without errors and generating the expected analytical outputs.

**Figure 1:**
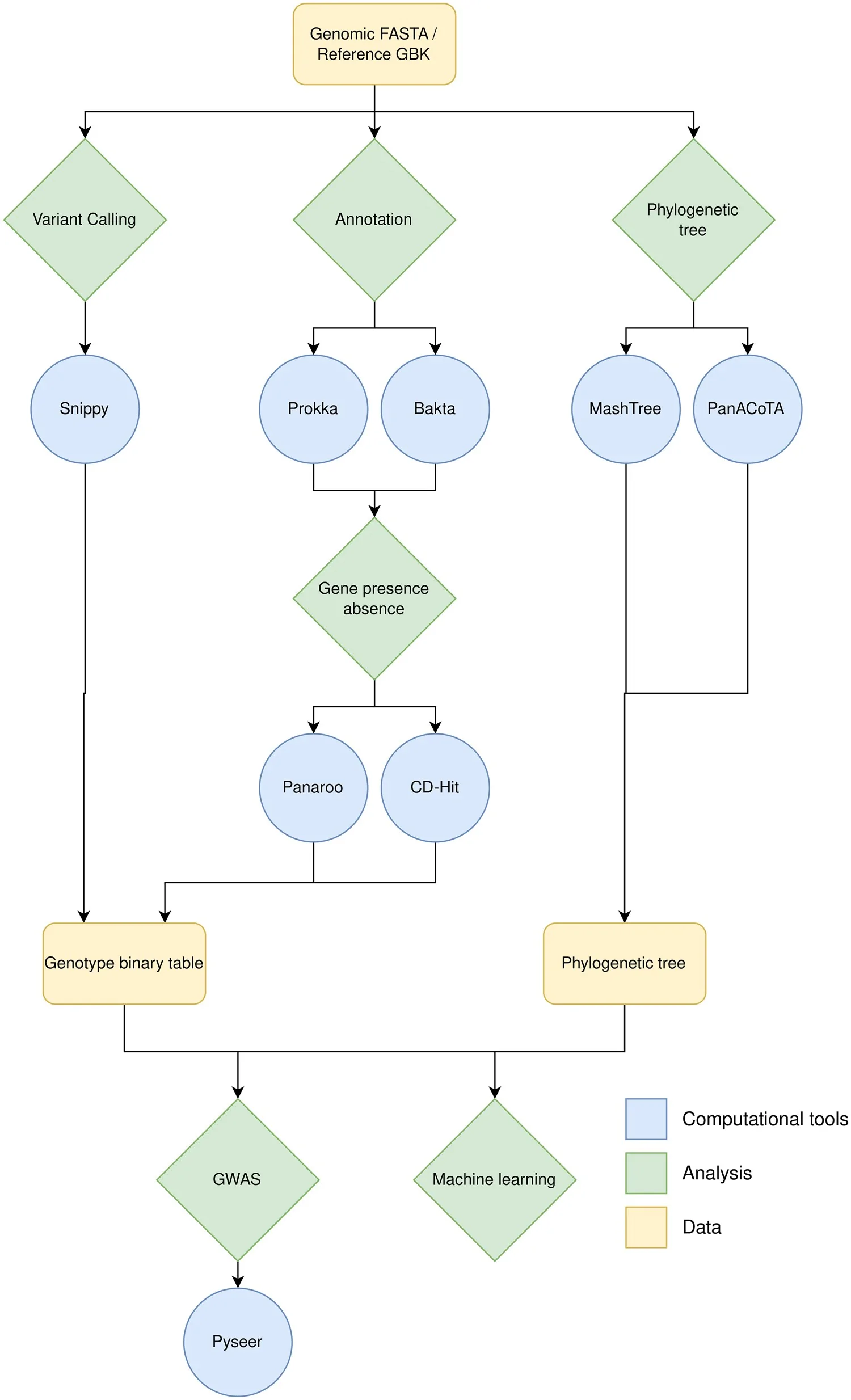
General overview of the ALPAR’s pipeline.

While ALPAR fully supports Bakta (Schwengers *et al.* 2021) for modern, actively curated annotations, Prokka (Seemann 2014) is retained as a highly optimized alternative (Fig. 1). Prokka consumes significantly less memory (∼2–3 GB vs. ∼12 GB per thread for Bakta (Schwengers *et al.* 2021)) and ensures native structural compatibility with downstream pan-genome tools like Panaroo, offering critical flexibility for users with constrained computational resources.

### 2.2 Use case: *Escherichia coli* AMR data from PATRIC database Wattam *et al.* (2017)

To showcase our approach, we applied the ALPAR pipeline to publicly available *E. coli* dataset of 1000 genomes (for the list see Supplementary File 4, available as supplementary data at *Bioinformatics* online) from the PATRIC database (Wattam *et al.* 2017). We selected *E. coli* as our example organism because it is a well-established bacterial model (Blount 2015). The genomes from the dataset were already annotated as resistant or susceptible; therefore, we did not need to perform any pre-preparation steps. We have used the automatix option to run the entire pipeline, as demonstrated by for both the Prokka (Seemann 2014) and Bakta (Schwengers *et al.* 2021) annotation tools:

# Configuration for a consumer-grade PC (e.g., Intel Core i7, 16GB RAM)

alpar automatix \

-i EcoliTestData/ -o ecoli_out_prokka \

--reference Escherichia_coli.gbff \

--annotation_tool prokka \

--prokka_custom_database Escherichia_coli.fasta EC \

--gene_presence_absence_analysis_tool cd-hit \

--threads 12 --ram 14 --keep_temp_files \

--ml_algorithm xgb --fast --run_qc

# Configuration for a high-performance server (e.g., 128 threads, 1TiB RAM)

alpar automatix -i EcoliTestData/ -o ecoli_out_bakta \

--reference Escherichia_coli.gbff \

--annotation_tool bakta \

--bakta_db bakta_db/baktadb \

--gene_presence_absence_analysis_tool cd-hit \

--threads 120 --ram 800 --keep_temp_files \

--ml_algorithm xgb --fast --run_qc

Running the full pipeline took ∼ 22 hours wall time with Bakta (Schwengers *et al.* 2021) for annotations (with Prokka (Seemann 2014)) it took ∼9 hours for same input data with the same settings) on a 2× AMD EPYC 7601 32-Core Processors (128 threads total) and 1TiB of RAM, running Linux (CentOS 8, kernel 4.18.0). For the same input, it took ∼26 hours with 12 threads and 14GB RAM on a consumer-grade PC with Intel(R) Core(TM) i7-10700 CPU @ 2.90 GHz and 16GB of RAM, running Linux (Rocky Linux 8.10). 64 strains out of 1000 were skipped because they did not pass the quality control (qc) step. 936 strains that had been screened against ciprofloxacin were used in later steps. The pipeline has generated ∼4 58 000 features. Because single-reference pipelines can introduce systematic skew in highly diverse bacterial populations, we quantitatively evaluated potential reference bias within this dataset using MUMmer’s dnadiff utility (Marçais *et al.* 2018). We conducted an independent Welch’s *t*-test to compare alignment metrics between the resistant (n=450) and susceptible (n=550) strains in the *E. coli* ciprofloxacin resistance dataset. The analysis revealed a baseline reference bias that preferentially penalized the susceptible group; resistant strains mapped significantly better to the *E. coli* reference genome than susceptible strains in terms of both reference coverage (20.56% vs. 13.27%, $p=1.60\times 10^{-7}$) and query alignment (28.74% vs. 22.63%, $p=3.98\times 10^{-7}$). Furthermore, susceptible strains proved significantly more divergent from the reference, accumulating an average of 98 022 SNPs compared to 77 714 SNPs in the resistant group ($p=1.36\times 10^{-21}$), though the average nucleotide identity across aligned regions remained comparable (95.95% vs. 97.08%, p=0.062). While these results confirm that the selected reference genome is genetically closer to our resistant population, ALPAR mitigates the worst effects of this bias by utilizing independent assemblies to capture pan-genomic diversity rather than directly mapping raw reads. Full alignment statistics are provided in Supplementary Table S6, available as supplementary data at *Bioinformatics* online.

As a result of the automatix pipeline, our XGBoost classification model achieved an MCC of 0.881. Feature importance analysis (FIA), conducted by means of the Gini feature importance routine, yielded many significant mutations: the top findings were the V85V synonymous mutation in the GyrA protein (DNA gyrase subunit A) with an importance value 0.229, the D87N missense mutation in the GyrA protein with an importance value 0.214, the S80I missense mutation in the ParC protein (DNA topoisomerase 4 subunit A) with an importance value 0.204, and the S83L missense mutation in the GyrA protein with an importance value 0.163. All mutations are well-known resistance determinants towards fluoroquinolone drugs, of which ciprofloxacin is an example (Malekian *et al.* 2023). Details of these results are in Supplementary File 1, Fig. 1, and Supplementary File 5, Figs. 5 and 8, available as supplementary data at *Bioinformatics* online.

To further investigate the mechanism of resistance, we analyzed the identified mutations with StructMAn 2.0 Web, a web-based tool that annotates non-synonymous single-nucleotide variants (nsSNVs) by analyzing their structural context within 3D protein structures, including interactions with other macromolecules and ligands (Gress *et al.* 2016, Yurtseven *et al.* 2025). All missense mutation positions are structurally classified as interacting with a ligand, which can explain the resistance. The V85V synonymous mutation in the GyrA protein was classified as a core mutation; however, further analysis revealed that it is located in close proximity to nucleic acid (for the figures see Supplementary File 1, Fig. 1, available as supplementary data at *Bioinformatics* online).

To demonstrate the advantage of this integrated approach, we benchmarked the pyseer GWAS module against our XGBoost framework on the *E. coli* dataset. Using identical stratified DataSAIL splits, the pyseer decision tree trained on significant GWAS hits achieved an MCC of 0.148, whereas the XGBoost model achieved an MCC of 0.881. This highlights ALPAR’s capacity to capture complex, non-linear genetic interactions that traditional linear GWAS frameworks may fail to resolve under strict population stratification. Detailed visualizations of these pyseer GWAS outputs, including the generated graphs and the baseline decision tree, are provided in Supplementary File 5, Figs. 3 and 4, available as supplementary data at *Bioinformatics* online.

### 2.3 Use case: CAMDA anti-microbial resistance prediction challenge—2024

In the 2024 CAMDA challenge CAMDA Organizing Committee (2024), we focused on building ML models to predict the AMR status of 1820 bacterial strains (for the list see Supplementary File 4, available as supplementary data at *Bioinformatics* online) that belong to seven different species (*Campylobacter jejuni*, *Campylobacter coli*, *Escherichia coli*, *Klebsiella pneumoniae*, *Neisseria gonorrhoeae*, *Pseudomonas aeruginosa*, *Salmonella enterica*). For these strains, the AMR status towards two drugs (meropenem, ciprofloxacin) was provided by the challenge organizers.

Data provided by the challenge organizers consisted of short-read sequences. Since ALPAR requires genomic FASTA files, we first assembled the genomes using SPAdes (Prjibelski *et al.* 2020). Afterward, we employed the automatix module of ALPAR utilizing the random forest method. For each bacterial–antibiotic pair in the dataset, two models were trained: one including all the features and one only using the top 30% of the features ranked according to their PRPS score that penalizes variants that are localized to a single branch on the strains’ phylogenetic tree. Predictions on the test set were generated using both models and yielded identical resistance status classifications across all 1820 test strains. The prediction results (Supplementary File 3, available as supplementary data at *Bioinformatics* online) were submitted to the CAMDA challenge, achieving a score of 83 out of 100, which was the winning score in the challenge.

### 2.4 Use case: CAMDA anti-microbial resistance prediction challenge—2025

In the 2025 CAMDA challenge CAMDA Organizing Committee (2025), we focused on building ML models to predict the AMR status of 5345 bacterial strains (for the list see Supplementary File 4, available as supplementary data at *Bioinformatics* online) that belong to nine different species (*Acinetobacter baumannii*, *Campylobacter jejuni*, *Escherichia coli*, *Klebsiella pneumoniae*, *Neisseria gonorrhoeae*, *Pseudomonas aeruginosa*, *Salmonella enterica*, *Staphylococcus aureus*, *Streptococcus pneumoniae*). For these strains, the AMR status towards two drugs (gentamicin, tetracycline) was provided by the challenge organizers.

As in the previous year, the challenge organizers provided short-read sequences. Because ALPAR requires genomic FASTA files, we first assembled the genomes using SPAdes (Prjibelski *et al.* 2020). We then applied ALPAR with both Random Forest (RF) and XGBoost (XGB) methods, using a similarity-aware data split with DataSAIL (Joeres *et al.* 2025). For each bacterial–antibiotic pair in the dataset, two separate models were trained: one using all available features, and another incorporating additional gene-cluster–centric *k*-mer data generated with panfeed (Sommer *et al.* 2023). Predictions for the test set were produced using best of these models. These prediction results (Supplementary File 3, available as supplementary data at *Bioinformatics* online) were submitted to the CAMDA Challenge, where they achieved a score of 84 out of 100, ranking as the third-best model (submitted under the name “Yurtseven—extra submission”). To check the impact of the similarity-aware data splitting, we submitted two additional model results that used only variant and GPA features: one trained with a random split and the other with a DataSAIL (Joeres *et al.* 2025) split. Although the DataSAIL-split model showed slightly lower validation performance than the randomly split model during our testing, it achieved 2% higher accuracy on the real-life test data, indicating that the random split suffered from data leakage.

### 2.5 Benchmarking and external validation

To ensure broad generalizability and assess performance against existing rule-based pipelines, ALPAR was benchmarked against AMRFinderPlus using independent external datasets from the BV-BRC database and the CABBAGE (Dickens *et al.* 2025) (Comprehensive Assessment of Bacterial-Based AMR prediction from Genotypes) dataset. The evaluation encompassed 80 distinct bacteria-antibiotic pairs across 11 species. While AMRFinderPlus performed well on well-characterized, SNP-driven resistance profiles (e.g. fluoroquinolones), ALPAR consistently outperformed the rule-based approach overall, particularly in cases involving novel or less-defined resistance mechanisms, or in species lacking dedicated curated databases (such as *Mycobacterium tuberculosis*). Across all 80 benchmarking pairs, ALPAR achieved a weighted, with the number of strains, average MCC of 0.726, compared to 0.563 for AMRFinderPlus (Supplementary Table S7, available as supplementary data at *Bioinformatics* online). This independent validation confirms ALPAR’s capacity to generalize across geographical datasets and diverse clinical species without relying on predefined resistance catalogs.

## 3 Conclusion

ALPAR is a scalable, Conda installable tool for AMR analysis, capable of processing thousands of genomes on both laptops and high-performance clusters. By integrating modern tools like PRPS and DataSAIL, ALPAR addresses critical challenges in bacterial machine learning, specifically reducing the impact of phylogenetic structure and information leakage (James *et al.* 2025, Yu *et al.* 2025). This allows researchers to better differentiate between lineage-specific markers and genuine causal variants, ensuring model robustness across diverse bacterial clades.

While ALPAR currently relies on a single reference genome for consistency, a standard approach validated in several studies (Khaledi *et al.* 2020, Ren *et al.* 2022) we acknowledge that multi-reference, graph-based approaches represent a significant future direction (Colquhoun *et al.* 2021).

Ultimately, the novelty of ALPAR rests in its highly integrated design, providing a frictionless framework to bridge the gap between raw genomic data and robust ML-based AMR predictions. By streamlining otherwise disjointed and complex bioinformatic workflows into a cohesive pipeline, ALPAR facilitates more standardized, reproducible, and effective research in the global effort to combat antimicrobial resistance.

## Supplementary Material

btag545_Supplementary_Data

## Data Availability

The tool underlying this article is available at https://github.com/kalininalab/ALPAR

## References

[btag545-B1] Anahtar MN , Yang JH, Kanjilal S. Applications of machine learning to the problem of antimicrobial resistance: an emerging model for translational research. J Clin Microbiol 2021;59:e0126020. 10.1128/JCM.01260-20. https://jcm.asm.org/content/early/2021/01/28/JCM.01260-20.abstract. 33536291 PMC8218744

[btag545-B2] Ardila CM , González-Arroyave D, Tobón S. Machine learning for predicting antimicrobial resistance in critical and high-priority pathogens: a systematic review considering antimicrobial susceptibility tests in real-world healthcare settings. PLoS One 2025;20:e0319460. 10.1371/journal.pone.0319460. 39999193 PMC11856330

[btag545-B3] Blount ZD. The unexhausted potential of *E. coli*. Elife 2015;4:e05826. 10.7554/elife.05826. PMC4373459 25807083

[btag545-B4] Breiman L. Random forests. Mach Learn 2001;45:5–32. 10.1023/a:1010933404324. https://link.springer.com/article/10.1023/a : 1010933404324.

[btag545-B5] CAMDA Organizing Committee. The CAMDA contest challenges, 2024. https://bipress.boku.ac.at/camda-play/the-camda-contest-challenges/ (2 June 2026, date last accessed).

[btag545-B6] CAMDA Organizing Committee. The CAMDA contest challenges, 2025. URL https://bipress.boku.ac.at/camda2025/ (29 July 2026, date last accessed)

[btag545-B7] CDC. Antibiotic Resistance Threats in the United States. Atlanta: Centers for Disease Control and Prevention, 2019.

[btag545-B8] Chindelevitch L , Jauneikaitea E, Wheeler N et al Applying data technologies to combat AMR: current status, challenges, and opportunities on the way forward. arXiv *(Cornell University)*, 10.48550/arxiv.2208.04683, Jul 2022, preprint: not peer reviewed.

[btag545-B9] Chen PE , Shapiro BJ. The advent of genome-wide association studies for bacteria. Curr Opin Microbiol 2015;25:17–24. 10.1016/j.mib.2015.03.002. 25835153

[btag545-B10] Chewapreecha C , Marttinen P, Croucher NJ et al Comprehensive identification of single nucleotide polymorphisms associated with beta-lactam resistance within pneumococcal mosaic genes. PLoS Genet 2014;10:e1004547. 10.1371/journal.pgen.1004547. 25101644 PMC4125147

[btag545-B11] Colquhoun RM , Hall MB, Lima L et al Pandora: nucleotide-resolution bacterial pan-genomics with reference graphs. Genome Biol 2021;22:267. 10.1186/s13059-021-02473-1. 34521456 PMC8442373

[btag545-B12] Cortes C , Vapnik V. Support-vector networks. Mach Learn 1995;20:273–97.

[btag545-B13] Dickens E , Derelle R, Beardmore R et al A comprehensive AMR genotype-phenotype database (cabbage). 2025. 10.1101/2025.11.12.688105. PMC13501133 42635122

[btag545-B14] Elgart M , Lyons G, Romero-Brufau S et al Non-linear machine learning models incorporating snps and prs improve polygenic prediction in diverse human populations. Commun Biol 2022;5:856–12. 10.1038/s42003-022-03812-z. https://www.nature.com/articles/s42003-022-03812-z. 35995843 PMC9395509

[btag545-B15] Farhat MR , Sultana R, Iartchouk O et al Genetic determinants of drug resistance in *Mycobacterium tuberculosis* and their diagnostic value. Am J Respir Crit Care Med 2016;194:621–30. 10.1164/rccm.201510-2091oc. 26910495 PMC5027209

[btag545-B16] Gress A , Ramensky V, Buch J et al StructMAn: annotation of single-nucleotide polymorphisms in the structural context. Nucleic Acids Res 2016;44:W463–8. 10.1093/nar/gkw364. 27150811 PMC4987916

[btag545-B17] Holmes AH , Moore LS, Sundsfjord A et al Understanding the mechanisms and drivers of antimicrobial resistance. Lancet 2016;387:176–87. 26603922 10.1016/S0140-6736(15)00473-0

[btag545-B18] James T , Williamson B, Tino P et al Whole-genome phenotype prediction with machine learning: open problems in bacterial genomics. Bioinformatics 2025;41:btaf206. 10.1093/bioinformatics/btaf206. 40581074 PMC12237507

[btag545-B19] Joeres R , Blumenthal DB, Kalinina OV. Data splitting to avoid information leakage with datasail. Nat Commun 2025;16:3337. 40199913 10.1038/s41467-025-58606-8 PMC11978981

[btag545-B20] Katz L , Griswold T, Morrison S et al Mashtree: a rapid comparison of whole genome sequence files. J Open Source Softw 2019;4:1762. 10.21105/joss.01762. PMC9380445 35978566

[btag545-B21] Khaledi A , Weimann A, Schniederjans M et al Predicting antimicrobial resistance in *Pseudomonas aeruginosa* with machine learning‐enabled molecular diagnostics. EMBO Mol Med 2020;12:e10264. 10.15252/emmm.201910264. 32048461 PMC7059009

[btag545-B22] Kim JI , Maguire F, Tsang KK et al Machine learning for antimicrobial resistance prediction: current practice, limitations, and clinical perspective. Clin Microbiol Rev 2022;35:e0017921. 10.1128/cmr.00179-21. 35612324 PMC9491192

[btag545-B23] Kulkarni SG , Green AG, Mann BC et al Convolutional neural networks quantify antibiotic resistance in Mycobacterium tuberculosis with diagnostic grade accuracy and predict treatment response. *Nat Communnature Communications* 2026;2026;17:2402–17. 10.1038/s41467-02672225-x PMC13315309 42031767

[btag545-B24] Logan LK , Weinstein RA. The epidemiology of carbapenem-resistant *Enterobacteriaceae*: the impact and evolution of a global menace. J Infect Dis 2017;215: S28–36. 10.1093/infdis/jiw282. https://academic.oup.com/jid/article/215/suppl_1/S28/3092084. 28375512 PMC5853342

[btag545-B25] Malekian N , Sainath S, Al-Fatlawi A et al Word-based GWAS harnesses the rich potential of genomic data for *E. coli* quinolone resistance. Front Microbiol 2023;14:1276332. 10.3389/fmicb.2023.1276332. 38152371 PMC10751334

[btag545-B26] Marçais G , Delcher AL, Phillippy AM et al MUMmer4: a fast and versatile genome alignment system. PLoS Comput Biol 2018;14:e1005944. 10.1371/journal.pcbi.1005944. 29373581 PMC5802927

[btag545-B27] McArthur AG , Waglechner N, Nizam F et al The comprehensive antibiotic resistance database. Antimicrob Agents Chemother 2013;57:3348–57. 10.1128/aac.00419-13. https://aac.asm.org/content/57/7/3348.short. 23650175 PMC3697360

[btag545-B28] Moradigaravand D , Palm M, Farewell A et al Prediction of antibiotic resistance in *Escherichia coli* from large-scale pan-genome data. PLoS Comput Biol 2018;14:e1006258. 10.1371/journal.pcbi.1006258. https://journals.plos.org/ploscompbiol/article? id=10.1371%2Fjournal.pcbi.1006258. 30550564 PMC6310291

[btag545-B29] Mosquera-Rendon J , Moreno-Herrera CX, Robledo J et al Genome-wide association studies (GWAS) approaches for the detection of genetic variants associated with antibiotic resistance: a systematic review. Microorganisms 2023;11:2866. 10.3390/microorganisms11122866. https://www.mdpi.com/2076-2607/11/12/2866. 38138010 PMC10745584

[btag545-B30] Nguyen M , Long SW, McDermott PF et al Using machine learning to predict antimicrobial mics and associated genomic features for nontyphoidal Salmonella. J Clin Microbiol 2019;57:e01591–18. 10.1128/JCM.01591-18. PMC6355527 30333126

[btag545-B31] O’Neill J. Tackling Drug-resistant Infections Globally: Final Report and Recommendations. London: Wellcome Trust, 2016.

[btag545-B32] Paredes-Gutierrez G , Perea-Jacobo R, Acosta-Mesa H-G et al Predicting drug resistance in mycobacterium tuberculosis: a machine learning approach to genomic mutation analysis. Diagnostics (Basel) 2025;15:279. 10.3390/diagnostics15030279. https://www.mdpi.com/2075-4418/15/3/279. 39941209 PMC11817661

[btag545-B33] Perrin A , Rocha EPC. PanACoTA: a modular tool for massive microbial comparative genomics. NAR Genom Bioinform 2021;3:lqaa106. 10.1093/nargab/lqaa106. https://academic.oup.com/nargab/article/3/1/lqaa106/6090162?login=true. 33575648 PMC7803007

[btag545-B34] Pesesky MW , Hussain T, Wallace M et al Evaluation of machine learning and rules-based approaches for predicting antimicrobial resistance profiles in gram-negative bacilli from whole genome sequence data. Front Microbiol 2016;7:1887. 10.3389/fmicb.2016.01887. 27965630 PMC5124574

[btag545-B35] Power RA , Parkhill J, de Oliveira T. Microbial genome-wide association studies: lessons from human GWAS. Nat Rev Genet 2017;18:41–50. 10.1038/nrg.2016.132. 27840430

[btag545-B36] Prjibelski A , Antipov D, Meleshko D et al Using SPAdes de novo assembler. Curr Protoc Bioinformatics 2020;70:e102. 10.1002/cpbi.102. 32559359

[btag545-B37] Ranjbar R , Sami M. Genetic investigation of beta-lactam associated antibiotic resistance among *Escherichia coli* strains isolated from water sources. Open Microbiol J 2017;11:203–10. 10.2174/1874285801711010203. 29151997 PMC5678241

[btag545-B38] Read TD , Massey RC. Characterizing the genetic basis of bacterial phenotypes using genome-wide association studies: a new direction for bacteriology. Genome Med 2014;6:109. 10.1186/s13073-014-0109-z. 25593593 PMC4295408

[btag545-B39] Ren Y , Chakraborty T, Doijad S et al Prediction of antimicrobial resistance based on whole-genome sequencing and machine learning. Bioinformatics 2022;38:325–34. 10.1093/bioinformatics/btab681. 34613360 PMC8722762

[btag545-B40] Sakagianni A , Koufopoulou C, Feretzakis G et al Using machine learning to predict antimicrobial resistance―a literature review. Antibiotics 2023;12:452. 10.3390/antibiotics12030452. https://www.mdpi.com/2079-6382/12/3/452. 36978319 PMC10044642

[btag545-B41] Sakagianni A , Koufopoulou C, Koufopoulos P et al Data-driven approaches in antimicrobial resistance: machine learning solutions. Antibiotics (Basel) 2024;13:1052. 10.3390/antibiotics13111052. https://www.mdpi.com/2079-6382/13/11/1052. 39596745 PMC11590962

[btag545-B42] Schwengers O , Jelonek L, Dieckmann MA et al Bakta: rapid and standardized annotation of bacterial genomes via alignment-free sequence identification. Microb Genom 2021;7:000685. 10.1099/mgen.0.000685. 34739369 PMC8743544

[btag545-B43] Seemann T. Prokka: rapid prokaryotic genome annotation. Bioinformatics 2014;30:2068–9. 10.1093/bioinformatics/btu153. 24642063

[btag545-B44] Seemann T. Snippy: rapid haploid variant calling and core SNP phylogeny. *GitHub.* github.com/tseemann/snippy. 2015.

[btag545-B45] Sommer H , Djamalova D, Galardini M. Reduced ambiguity and improved interpretability of bacterial genome-wide associations using gene-cluster-centric *k*-mers. Microb Genom 2023;9:001129. 10.1099/mgen.0.001129. 37934071 PMC10711318

[btag545-B46] Tang R , Luo R, Tang S et al Machine learning in predicting antimicrobial resistance: a systematic review and meta-analysis. Int J Antimicrob Agents 2022;60:106684. 10.1016/j.ijantimicag.2022.106684. 36279973

[btag545-B47] Tonkin-Hill G , MacAlasdair N, Ruis C et al Producing polished prokaryotic pangenomes with the Panaroo pipeline. Genome Biol 2020;21:180. 10.1186/s13059-020-02090-4. 32698896 PMC7376924

[btag545-B48] Ventola CL. The antibiotic resistance crisis: part 1: causes and threats. P T 2015;40:277–83. 25859123 PMC4378521

[btag545-B49] Wattam AR , Davis JJ, Assaf R et al Improvements to patric, the all-bacterial bioinformatics database and analysis resource center. Nucleic Acids Res 2017;45:D535–42. 10.1093/nar/gkw1017. 27899627 PMC5210524

[btag545-B50] Weber RE , Fuchs S, Layer F et al Genome-wide association studies for the detection of genetic variants associated with Daptomycin and Ceftaroline resistance in *Staphylococcus aureus*. Front Microbiol 2021;12:686197. 10.3389/fmicb.2021.639660. 33986737 PMC8111692

[btag545-B51] World Health Organization. *Antimicrobial Resistance: Global Report on Surveillance.* Geneva: World Health Organization, 2014.

[btag545-B52] Wu Y-W , Chen Y-T, Chang Y-H et al Enhancing predictions of antimicrobial resistance of pathogens by expanding the potential resistance gene repertoire using a pan-genome-based feature selection approach. BMC Bioinformatics 2022;23:1–17. 10.1186/s12859-022-04666-2. 35428201 PMC9011928

[btag545-B53] Yang Y , Walker TM, Walker AS et al Deepamr for predicting co-occurrent resistance of *Mycobacterium tuberculosis*. Bioinformatics 2019;35:3240–9. 10.1093/bioinformatics/btz067. 30689732 PMC6748723

[btag545-B54] Yu Y , Wheeler NE, Barquist L. Biased sampling driven by bacterial population structure confounds machine learning prediction of antimicrobial resistance. PLoS Biol 2025;23:e3003539. 10.1371/journal.pbio.3003539. 41401143 PMC12707637

[btag545-B55] Yurtseven A , Buyanova S, Agrawal AA et al Machine learning and phylogenetic analysis allow for predicting antibiotic resistance in *M. tuberculosis*. BMC Microbiol 2023;23:404. 10.1186/s12866-023-03147-7. 38124060 PMC10731705

[btag545-B56] Yurtseven A , Keller S, Hirsch P et al Structman 2.0 web: a web server for structural annotation of protein sequences and mutations. Nucleic Acids Res 2025;53:W528–33. 10.1093/nar/gkaf381. https://pubmed.ncbi.nlm.nih.gov/40326516/. 40326516 PMC12230719

[btag545-B57] Zankari E , Hasman H, Cosentino S et al Identification of acquired antimicrobial resistance genes. J Antimicrob Chemother 2012;67:2640–4. 10.1093/jac/dks261. 22782487 PMC3468078

[btag545-B58] Zou Z , Tang F, Qiao L et al Integrating sequencing methods with machine learning for antimicrobial susceptibility testing in pediatric infections: current advances and future insights. Front Microbiol 2025;16:1528696. 10.3389/fmicb.2025.1528696. 40109965 PMC11919855

