# Supplementary material for: ALPAR: automated learning pipeline for antimicrobial resistance
Source: Bioinformatics. 2026 Jul 22;42(8):btag545. doi: 10.1093/bioinformatics/btag545 (PMC13437014; doi:10.1093/bioinformatics/btag545)
Supplement: Supplementary file 1 [file btag545_supplementary_data.zip › supplementary2.pdf]

# Supplementary 2. Example of binary genotype and phenotype tables

Table 1: Binary genotype table with variants and gene presence-absence example

| Strain | ’2932974’, ’G:A’, ’snp’ | ’5185038’, ’T:C’, ’snp’ | ’4552768’, ’G:A’, ’snp’ | ’4775234’, ’G:A’, ’snp’ | ’2064043’, ’C:T’, ’snp’ | ’1039567’, ’C:T’, ’snp’ | ’3411705’,’G:GAA’,’ins’ | ’1955768’, ’C:T’, ’snp’ | ’4252620’,’GA:G’,’del’ | ’388184’, ’T:C’, ’snp’ | 0AKAZYY4_NNDNEAKK_00376_sodium_sulfate_symporter | 0AKAZYY4_NNDNEAKK_01823_asparagine_synthetase | 0AKAZYY4_NNDNEAKK_05043_phenazine_modifying_protein | JCKSVQIA_FNCGEDBI_05713_copper_resistance_protein_A | 0AKAZYY4_NNDNEAKK_01948_chaperone |
|---|---|---|---|---|---|---|---|---|---|---|---|---|---|---|---|
| Strain_1 | 0 | 1 | 0 | 1 | 1 | 1 | 0 | 1 | 1 | 1 | 0 | 1 | 1 | 0 | 0 |
| Strain_2 | 1 | 1 | 1 | 0 | 0 | 0 | 0 | 1 | 0 | 1 | 1 | 0 | 0 | 0 | 1 |
| Strain_3 | 0 | 1 | 1 | 0 | 1 | 0 | 0 | 1 | 0 | 1 | 0 | 1 | 0 | 0 | 0 |
| Strain_4 | 0 | 1 | 0 | 1 | 0 | 1 | 1 | 0 | 1 | 1 | 0 | 0 | 0 | 1 | 0 |
| Strain_5 | 0 | 1 | 1 | 1 | 1 | 0 | 0 | 0 | 1 | 1 | 1 | 0 | 1 | 0 | 1 |

Table 2: Binary phenotype table

| Strain | **Ciprofloxacin** | **Amikacin** | **Ethionamide** |
|---|---|---|---|
| Strain_1 | 0 | 1 | 0 |
| Strain_2 | 1 | 1 | 1 |
| Strain_3 | 0 | 1 | 1 |
| Strain_4 | 0 | 1 | 0 |
| Strain_5 | 0 | 1 | 1 |
