# Supplementary material for: ALPAR: automated learning pipeline for antimicrobial resistance
Source: Bioinformatics. 2026 Jul 22;42(8):btag545. doi: 10.1093/bioinformatics/btag545 (PMC13437014; doi:10.1093/bioinformatics/btag545)
Supplement: Supplementary file 1 [file btag545_supplementary_data.zip › supplementary1.pdf]

Supplementary 1. Use case: *Escherichia coli* AMR data from PATRIC Database feature importance results visualization

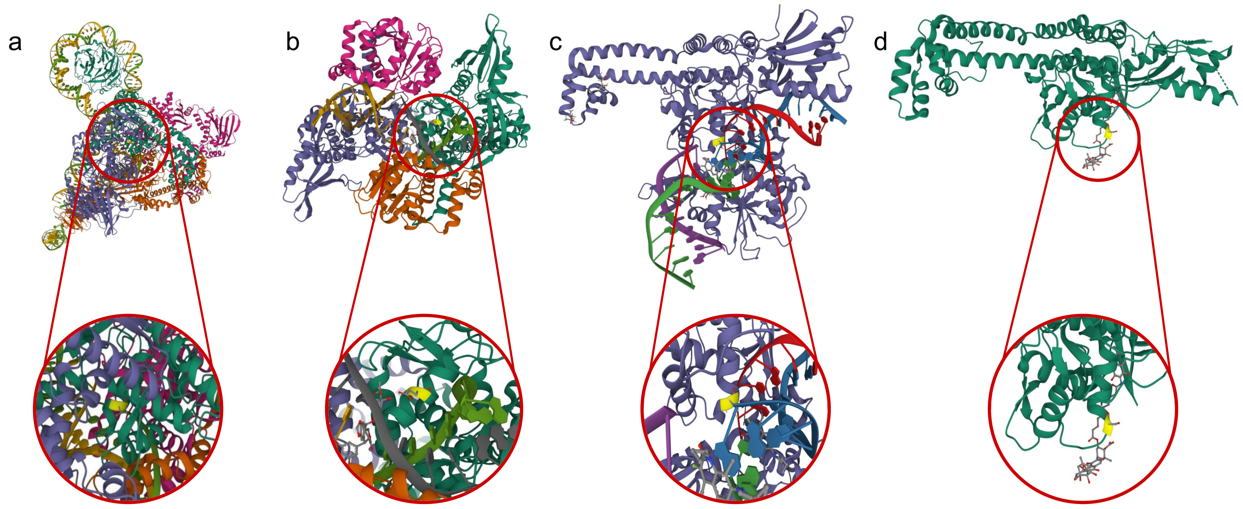

Figure 1: Mapping of mutations with the weights 0.452, 0.288, 0.091 and 0.032 in the FIA for the *E. coli* dataset. (a) The V85V mutation in GyrA; mapping based on the experimentally resolved structure of the *E. coli* GyrA, PDB ID: 9GBV. (b) The D87N mutation in GyrA; mapping based on the experimentally resolved structure of the *S.aureus* GyrA, PDB ID: 5CDP. (c) The S83L mutation in GyrA; mapping based on the experimentally resolved structure of the *E. coli* GyrA, PDB ID: 4CKL. (d) The S80I mutation in ParC; mapping based on the experimentally resolved structure of the *P. putida* ParC, PDB ID: 6BQ9. Mutated amino acids are shown in yellow and visualized using StructMAn Web [1]

.

References

[1] Alper Yurtseven, Sebastian Keller, Pascal Hirsch, Olga V Kalinina, and Alexander Gress. Structman 2.0 web: a web server for structural annotation of protein sequences and mutations. *Nucleic acids research*, page gkaf381, Jun 2025.
