# Supplementary material for: ALPAR: automated learning pipeline for antimicrobial resistance
Source: Bioinformatics. 2026 Jul 22;42(8):btag545. doi: 10.1093/bioinformatics/btag545 (PMC13437014; doi:10.1093/bioinformatics/btag545)
Supplement: Supplementary file 1 [file btag545_supplementary_data.zip › supplementary3.pdf]

Supplementary 3. CAMDA 2024 Results: Model Performance and Hyperparameters for XGBoost models, splitted with DataSAIL

Klebsiella pneumoniae – meropenem

MCC: 0.6863978564506099
Parameters: max_depth=7, min_child_weight=3, subsample=1.0, colsample_bytree=1.0, eta=0.01, n_estimators=100

Escherichia coli – meropenem

MCC: 0.4055175020198813
Parameters: max_depth=3, min_child_weight=1, subsample=0.6, colsample_bytree=0.8, eta=0.1, n_estimators=50

Salmonella enterica – ciprofloxacin

MCC: 0.0
Parameters: max_depth=3, min_child_weight=1, subsample=0.6, colsample_bytree=0.6, eta=0.01, n_estimators=50

Campylobacter jejuni – ciprofloxacin

MCC: 1.0
Parameters: max_depth=3, min_child_weight=1, subsample=0.6, colsample_bytree=0.6, eta=0.01, n_estimators=100

Acinetobacter baumannii – meropenem

MCC: 0.866524697803308
Parameters: max_depth=3, min_child_weight=5, subsample=0.6, colsample_bytree=0.8, eta=0.1, n_estimators=50

Neisseria gonorrhoeae – ciprofloxacin

MCC: 0.8958064164776167
Parameters: max_depth=3, min_child_weight=1, subsample=0.6, colsample_bytree=0.6, eta=0.01, n_estimators=200

Enterobacter cloacae – meropenem

MCC: 0.7142857142857143
Parameters: max_depth=3, min_child_weight=3, subsample=0.6, colsample_bytree=0.6, eta=0.2, n_estimators=50

Pseudomonas aeruginosa – meropenem

MCC: 0.5632113884748222
Parameters: max_depth=5, min_child_weight=5, subsample=0.6, colsample_bytree=1.0, eta=0.2, n_estimators=200

CAMDA 2025 Results: Model Performance and Hyperparameters for XGBoost models, splitted with DataSAIL

Neisseria gonorrhoeae – TET

MCC: 0.5431540161542693
Parameters: max_depth=3, min_child_weight=1, subsample=0.8, colsample_bytree=0.8, eta=0.01, n_estimators=500

Salmonella enterica – GEN

MCC: 0.9159481639050536
Parameters: max_depth=6, min_child_weight=5, subsample=0.8, colsample_bytree=0.8, eta=0.1, n_estimators=500

Pseudomonas aeruginosa – CAZ

MCC: 0.0
Parameters: max_depth=3, min_child_weight=1, subsample=0.8, colsample_bytree=0.8, eta=0.01, n_estimators=500

Acinetobacter baumannii – CAZ

MCC: 0.6154876417636101
Parameters: max_depth=3, min_child_weight=1, subsample=0.8, colsample_bytree=0.8, eta=0.05, n_estimators=1000

Campylobacter jejuni – TET

MCC: 0.9309931198210888
Parameters: max_depth=3, min_child_weight=1, subsample=0.8, colsample_bytree=0.8, eta=0.01, n_estimators=500

Streptococcus pneumoniae – ERY

MCC: 0.944606427885211
Parameters: max_depth=3, min_child_weight=1, subsample=0.8, colsample_bytree=0.8, eta=0.01, n_estimators=500

Staphylococcus aureus – ERY

MCC: 0.8166659366742163
Parameters: max_depth=3, min_child_weight=1, subsample=0.8, colsample_bytree=0.8, eta=0.01, n_estimators=500

Klebsiella pneumoniae – GEN

MCC: 0.5774599667671957
Parameters: max_depth=3, min_child_weight=1, subsample=0.8, colsample_bytree=0.8, eta=0.01, n_estimators=500

Escherichia coli – GEN

MCC: 0.4327156090943675
Parameters: max_depth=6, min_child_weight=1, subsample=0.8, colsample_bytree=0.8, eta=0.01, n_estimators=500
