# Supplementary material for: ALPAR: automated learning pipeline for antimicrobial resistance
Source: Bioinformatics. 2026 Jul 22;42(8):btag545. doi: 10.1093/bioinformatics/btag545 (PMC13437014; doi:10.1093/bioinformatics/btag545)
Supplement: Supplementary file 1 [file btag545_supplementary_data.zip › supplementary5.pdf]

Supplementary 5. Example results.

QC Results

strain	path	passed	reason
562.24650.fna	EcoliTestData/ciprofloxacin/Resistant/562.24650.fna	False	Length 95850 is outside bounds [4599751, 5621917]
562.24651.fna	EcoliTestData/ciprofloxacin/Resistant/562.24651.fna	False	Length 49828 is outside bounds [4599751, 5621917]
562.24654.fna	EcoliTestData/ciprofloxacin/Resistant/562.24654.fna	False	Length 46161 is outside bounds [4599751, 5621917]
562.24652.fna	EcoliTestData/ciprofloxacin/Resistant/562.24652.fna	False	Length 84929 is outside bounds [4599751, 5621917]
562.24653.fna	EcoliTestData/ciprofloxacin/Resistant/562.24653.fna	False	Length 163045 is outside bounds [4599751, 5621917]
562.24655.fna	EcoliTestData/ciprofloxacin/Resistant/562.24655.fna	False	Length 50228 is outside bounds [4599751, 5621917]
562.24656.fna	EcoliTestData/ciprofloxacin/Resistant/562.24656.fna	False	Length 9396 is outside bounds [4599751, 5621917]
562.24657.fna	EcoliTestData/ciprofloxacin/Resistant/562.24657.fna	False	Length 114267 is outside bounds [4599751, 5621917]
562.24658.fna	EcoliTestData/ciprofloxacin/Resistant/562.24658.fna	False	Length 85195 is outside bounds [4599751, 5621917]
562.24659.fna	EcoliTestData/ciprofloxacin/Resistant/562.24659.fna	False	Length 31765 is outside bounds [4599751, 5621917]
562.24798.fna	EcoliTestData/ciprofloxacin/Resistant/562.24798.fna	False	Length 181436 is outside bounds [4599751, 5621917]
562.24660.fna	EcoliTestData/ciprofloxacin/Resistant/562.24660.fna	False	Length 117703 is outside bounds [4599751, 5621917]
562.24661.fna	EcoliTestData/ciprofloxacin/Resistant/562.24661.fna	False	Length 46159 is outside bounds [4599751, 5621917]
562.24662.fna	EcoliTestData/ciprofloxacin/Resistant/562.24662.fna	False	Length 50235 is outside bounds [4599751, 5621917]
562.24663.fna	EcoliTestData/ciprofloxacin/Resistant/562.24663.fna	False	Length 216378 is outside bounds [4599751, 5621917]
562.24664.fna	EcoliTestData/ciprofloxacin/Resistant/562.24664.fna	False	Length 128094 is outside bounds [4599751, 5621917]
562.24672.fna	EcoliTestData/ciprofloxacin/Resistant/562.24672.fna	False	Length 169449 is outside bounds [4599751, 5621917]
562.23100.fna	EcoliTestData/ciprofloxacin/Resistant/562.23100.fna	True	Pass
562.23108.fna	EcoliTestData/ciprofloxacin/Resistant/562.23108.fna	True	Pass
562.23117.fna	EcoliTestData/ciprofloxacin/Resistant/562.23117.fna	True	Pass
562.23134.fna	EcoliTestData/ciprofloxacin/Resistant/562.23134.fna	True	Pass
562.23141.fna	EcoliTestData/ciprofloxacin/Resistant/562.23141.fna	True	Pass
562.23144.fna	EcoliTestData/ciprofloxacin/Resistant/562.23144.fna	True	Pass
562.23159.fna	EcoliTestData/ciprofloxacin/Resistant/562.23159.fna	True	Pass
562.24622.fna	EcoliTestData/ciprofloxacin/Resistant/562.24622.fna	False	Length 107210 is outside bounds [4599751, 5621917]
562.24623.fna	EcoliTestData/ciprofloxacin/Resistant/562.24623.fna	False	Length 111212 is outside bounds [4599751, 5621917]
562.24673.fna	EcoliTestData/ciprofloxacin/Resistant/562.24673.fna	False	Length 101165 is outside bounds [4599751, 5621917]
562.24624.fna	EcoliTestData/ciprofloxacin/Resistant/562.24624.fna	False	Length 51515 is outside bounds [4599751, 5621917]
562.24629.fna	EcoliTestData/ciprofloxacin/Resistant/562.24629.fna	False	Length 154099 is outside bounds [4599751, 5621917]
562.24630.fna	EcoliTestData/ciprofloxacin/Resistant/562.24630.fna	False	Length 60961 is outside bounds [4599751, 5621917]
562.24631.fna	EcoliTestData/ciprofloxacin/Resistant/562.24631.fna	False	Length 135720 is outside bounds [4599751, 5621917]
562.24644.fna	EcoliTestData/ciprofloxacin/Resistant/562.24644.fna	False	Length 48528 is outside bounds [4599751, 5621917]
562.24645.fna	EcoliTestData/ciprofloxacin/Resistant/562.24645.fna	False	Length 46161 is outside bounds [4599751, 5621917]
562.24646.fna	EcoliTestData/ciprofloxacin/Resistant/562.24646.fna	False	Length 244955 is outside bounds [4599751, 5621917]
562.24647.fna	EcoliTestData/ciprofloxacin/Resistant/562.24647.fna	False	Length 97474 is outside bounds [4599751, 5621917]
562.24648.fna	EcoliTestData/ciprofloxacin/Resistant/562.24648.fna	False	Length 64372 is outside bounds [4599751, 5621917]
562.24649.fna	EcoliTestData/ciprofloxacin/Resistant/562.24649.fna	False	Length 23332 is outside bounds [4599751, 5621917]
562.23167.fna	EcoliTestData/ciprofloxacin/Resistant/562.23167.fna	True	Pass
562.23181.fna	EcoliTestData/ciprofloxacin/Resistant/562.23181.fna	True	Pass
562.23307.fna	EcoliTestData/ciprofloxacin/Resistant/562.23307.fna	True	Pass
562.23191.fna	EcoliTestData/ciprofloxacin/Resistant/562.23191.fna	True	Pass
562.23194.fna	EcoliTestData/ciprofloxacin/Resistant/562.23194.fna	True	Pass
562.23195.fna	EcoliTestData/ciprofloxacin/Resistant/562.23195.fna	True	Pass
562.23199.fna	EcoliTestData/ciprofloxacin/Resistant/562.23199.fna	True	Pass
562.23200.fna	EcoliTestData/ciprofloxacin/Resistant/562.23200.fna	True	Pass
562.23205.fna	EcoliTestData/ciprofloxacin/Resistant/562.23205.fna	True	Pass

Figure 1: Example of quality control report. Showing strain, path, passed(True or False), reason in tab seperated format (tsv).

GWAS

```
variant af	filter-pvalue	lrt-pvalue	beta	beta-std-err	variant_h2	notes
'2339166','G:A','snp'	5.64E-01	1.98E-177	1.57E-88	-7.12E-01	3.20E-02	5.89E-01
'2339162','CATAG:TATAA','complex'	3.80E-01	7.64E-159	3.25E-73	6.54E-01	3.30E-02	5.44E-01
'3165734','GC:AA','complex'	2.56E-01	2.14E-92	3.50E-33	7.98E-01	6.39E-02	3.78E-01
'3165735','C:A','snp'	1.31E-01	5.00E-41	2.95E-31	5.97E-01	4.95E-02	3.67E-01
'1184408','C:T','snp'	2.36E-01	5.11E-84	4.50E-30	9.81E-01	8.31E-02	3.60E-01
'1909857','A:G','snp'	2.36E-01	5.11E-84	4.50E-30	9.81E-01	8.31E-02	3.60E-01
'2525498','CGTG:TGTA','complex'	2.36E-01	5.11E-84	4.50E-30	9.81E-01	8.31E-02	3.60E-01
'3078433','G:A','snp'	2.36E-01	5.11E-84	4.50E-30	9.81E-01	8.31E-02	3.60E-01
'3234731','TCC:CCT','complex'	2.36E-01	5.11E-84	4.50E-30	9.81E-01	8.31E-02	3.60E-01
'3254435','C:T','snp'	2.36E-01	5.11E-84	4.50E-30	9.81E-01	8.31E-02	3.60E-01
'4044328','A:T','snp'	2.36E-01	5.11E-84	4.50E-30	9.81E-01	8.31E-02	3.60E-01
'977003','CGGCGTGCGT:TGGGGTGTGG','complex'	2.36E-01	5.11E-84	4.50E-30	9.81E-01	8.31E-02	3.60E-01
'3165734','G:A','snp'	4.07E-01	8.79E-101	2.39E-25	-5.58E-01	5.21E-02	3.31E-01
'2525498','C:T','snp'	4.82E-01	1.02E-93	1.05E-24	-8.04E-01	7.61E-02	3.27E-01
'1495905','C:A','snp'	2.35E-01	1.61E-83	8.66E-24	7.82E-01	7.57E-02	3.20E-01
'1501299','C:T','snp'	2.35E-01	1.61E-83	8.66E-24	7.82E-01	7.57E-02	3.20E-01
'2449001','GCAC:TCAT','complex'	1.59E-01	9.30E-01	6.62E-23	8.99E-01	8.88E-02	3.14E-01
'977003','CGGC:TGGG','complex'	2.84E-01	8.25E-54	6.64E-23	-7.45E-01	7.36E-02	3.14E-01
'2339173','G:A','snp'	4.72E-01	2.20E-155	8.16E-23	2.44E-01	2.42E-02	3.14E-01
'2448968','T:C','snp'	2.76E-01	3.81E-04	1.02E-22	8.54E-01	8.48E-02	3.13E-01
'395370','A:G','snp'	8.12E-02	1.22E-01	2.02E-22	9.32E-01	9.32E-02	3.11E-01
'2448788','G:A','snp'	1.57E-01	9.51E-01	3.95E-22	8.76E-01	8.83E-02	3.09E-01
'93969','C:T','snp'	3.31E-02	5.41E-02	4.79E-22	-9.19E-01	9.28E-02	3.08E-01
'2922498','G:GCACCGCTAAAACGGCATTTA','ins'	2.78E-02	6.65E-05	5.85E-22	9.07E-01	9.18E-02	3.08E-01
'977012','T:G','snp'	5.82E-01	1.89E-97	5.35E-21	-6.75E-01	7.01E-02	3.01E-01
'362300','G:C','snp'	2.41E-01	9.28E-79	9.99E-21	7.33E-01	7.67E-02	2.99E-01
'3598428','T:C','snp'	3.95E-02	8.38E-03	2.79E-20	-9.05E-01	9.58E-02	2.95E-01
```

Figure 2: Example of GWAS results sorted according to their significance in tab seperated format (tsv). Later this results are used to generate graph (see. Figure3).

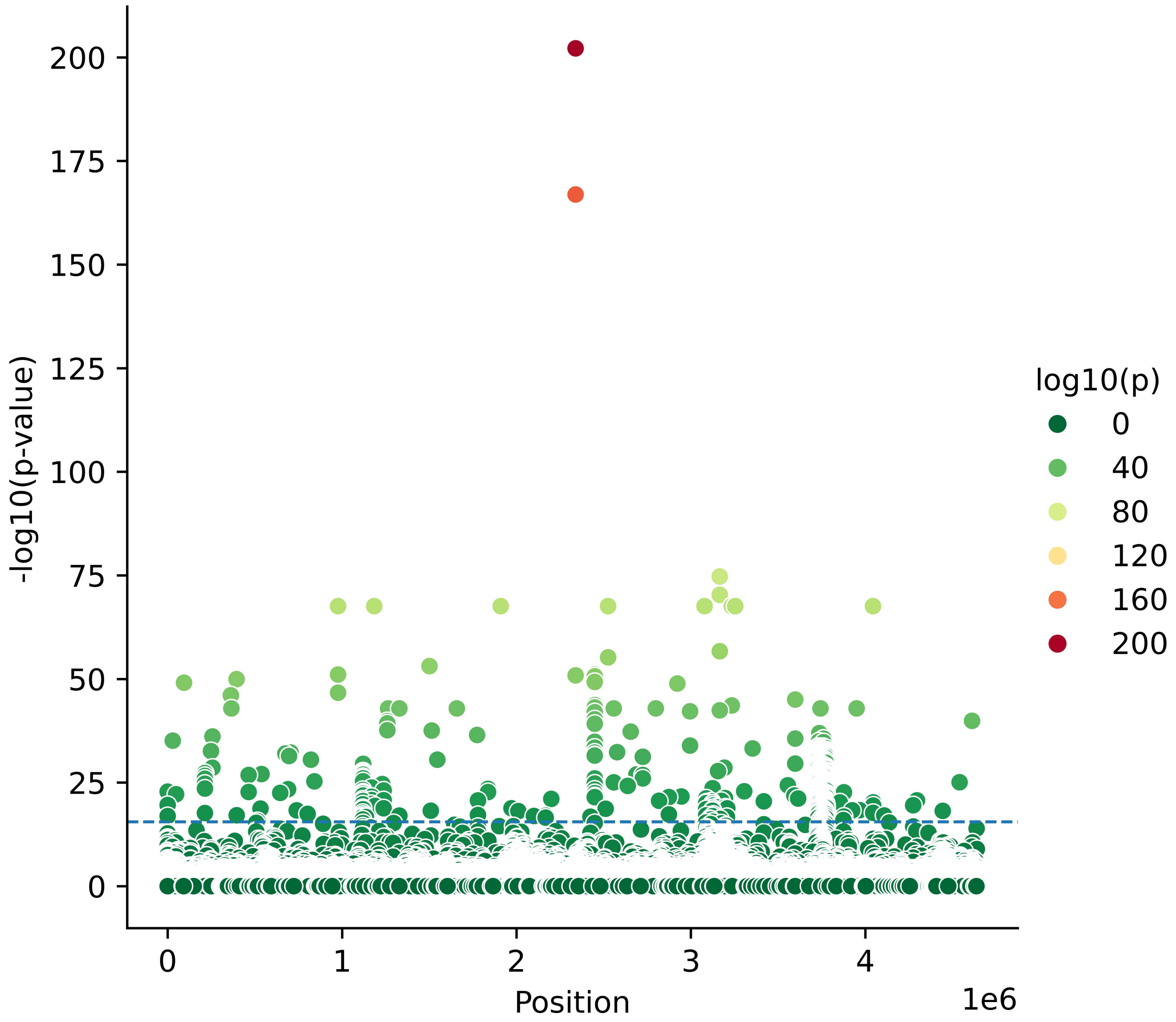

Figure 3: Example of GWAS results graph. Each data point shows mutation. Y axis represent significance (green: less significant, red: more significant). X axis represents mutation position on reference genome. Horizontal dashed line shows Bonferroni corrected significance threshold.

```
Accuracy score: 0.5654761904761905
Balanced Accuracy score: 0.5811864406779661
Matthews correlation coefficient: 0.14848787571052333
F1 score binary: 0.6368159203980099
Precision score: 0.7710843373493976
Recall score: 0.5423728813559322
Confusion matrix:
[[31 19]
 [54 64]]
ROC AUC Score: 0.5811864406779661
Brier score loss: 0.43452380952380953
Jaccard score: 0.46715328467153283
Log loss: 15.661825579794952
```

Figure 4: Example of GWAS decision tree results. Trained with using only GWAS significant mutations.

ML

```
Accuracy score: 0.9464285714285714
Balanced Accuracy score: 0.9561016949152542
Matthews correlation coefficient: 0.880884098694234
F1 score binary: 0.9606986899563319
Precision score: 0.990990990990991
Recall score: 0.9322033898305084
Confusion matrix:
[[ 49   1]
 [  8 110]]
ROC AUC Score: 0.9561016949152542
Brier score loss: 0.05357142857142857
Jaccard score: 0.9243697478991597
Log loss: 1.9309100029884187
```

Figure 5: Example of ML results.

```
ML algorithm: xgb
Scoring method: MCC
Test train split: 0.2
Random state: 42
Number of threads: 32
Amount of ram: 600
Feature importance analysis: True
Feature importance analysis strategy: gini
Number of repeats for feature importance analysis: 5
DataSAIL distance matrix used for data split: None
DataSAIL epsilon used for data split: 0.1
DataSAIL delta used for data split: 0.1
DataSAIL solver used for data split: SCIP
DataSAIL max time used for data split: 600
DataSAIL stratify used for data split: False
```

Figure 6: Example of ML results used parameters.

```
Best MCC result for ciprofloxacin: 0.880884098694234
Parameters: max_depth=3, min_child_weight=3, subsample=1.0, colsample_bytree=0.6, eta=0.01, n_estimators=50
```

Figure 7: Example of ML results best model parameters.

```
Mutation;ImportanceValue;Effect;Gene;Product
'2339166','G:A','snp';0.22985626757144928;synonymous_variant c.255C>T p.Val85Val;gyrA;DNA gyrase subunit A
'2339162','CATAG:TATAA','complex';0.21475109457969666;missense_variant c.255_259delCTATGinsTTATA p.Asp87Asn;gyrA;DNA gyrase subunit A
'3165734','GC:AA','complex';0.2045256346464157;missense_variant c.239_240delGCinsTT p.Ser80Ile;parC;DNA topoisomerase IV subunit A
'2339173','G:A','snp';0.16316930949687958;missense_variant c.248C>T p.Ser83Leu;gyrA;DNA gyrase subunit A
'4451582','T:C','snp';0.03700537979602814;synonymous_variant c.525T>C p.Ser175Ser;ytfR;galactofuranose ABC transporter putative ATP binding subunit
'2377198','A:G','snp';0.031465139240026474;missense_variant c.395T>C p.Val132Ala;menH;2-succinyl-6-hydroxy-2, 4-cyclohexadiene-1-carboxylate synthase
'3300111','T:C','snp';0.02903584949672222;missense_variant c.146T>C p.Val49Ala;yhbQ;DNA damage response nuclease YhbQ
'3165734','G:A','snp';0.01806274801492691;synonymous_variant c.240C>T p.Ser80Ser;parC;DNA topoisomerase IV subunit A
'3024084','A:C','snp';0.016654061153531075;synonymous_variant c.2769A>C p.Ala923Ala;xdhD;fused putative xanthine/hypoxanthine oxidase: molybdopterin-binding subunit and Fe-S binding subunit
'3165735','C:A','snp';0.01574198342859745;missense_variant c.239G>T p.Ser80Ile;parC;DNA topoisomerase IV subunit A
'3101381','G:A','snp';0.007179133128374815;synonymous_variant c.243C>T p.Arg81Arg;yggN;DUF2884 domain-containing protein YggN
'3167566','C:T','snp';0.006900184787809849;synonymous_variant c.153G>A p.Val51Val;ygiS;putative deoxycholate binding periplasmic protein
'977003','CGGC:TGGG','complex';0.004372722003608942;synonymous_variant c.678_681delCGGCinsTGGG p.228;mukB;chromosome partitioning protein MukB
'4453138','A:C','snp';0.004116183612495661;missense_variant c.568A>C p.Ile190Leu;ytfT;galactofuranose ABC transporter putative membrane subunit YtfT
'3175196','A:C','snp';0.002885933266952634;synonymous_variant c.201T>G p.Val67Val;parE;DNA topoisomerase IV subunit B
'1993345','A:G','snp';0.002810501493513584;synonymous_variant c.1362T>C p.Ile454Ile;uvrC;UvrABC excision nuclease subunit C
'2027649','A:G','snp';0.0024174328427761793;synonymous_variant c.369T>C p.Thr123Thr;dgcQ;diguanylate cyclase DgcQ
'2344803','GT:TC','complex';0.002150828018784523;;;
'146858','G:C','snp';0.001991100376471877;;;
'1997296','T:C','snp';0.0007652100757695735;synonymous_variant c.519A>G p.Ser173Ser;tcyN;cystine ABC transporter ATP binding subunit
'3122038','GCGCC:CCACT','complex';0.0005068655591458082;missense_variant c.1764_1768delGGCGCinsAGTGG p.AlaGln589ValGlu;glcB;malate synthase G
'3121991','C:A','snp';0.0004504827957134694;synonymous_variant c.1815G>T p.Gly605Gly;glcB;malate synthase G
'1416944','C:T','snp';0.0004281830915715545 4;missense_variant c.443G>A p.Gly148Glu;recE;exonuclease VIII, ds DNA exonuclease, 5' --> 3' specific
'2474502','G:A','snp';0.0003204822423867881 3;synonymous_variant c.471G>A p.Gln157Gln;yfdQ;DUF2303 domain-containing protein YfdQ
'2146101','T:G','snp';0.0002970660862047225;missense_variant c.2836T>G p.Ser946Ala;dgcE;putative diguanylate cyclase DgcE
```

Figure 8: Example of ML results annotated FIA analysis.
